# Supplementary material for: A wind-albedo-wind feedback driven by landscape evolution
Source: Nat Commun. 2020 Jan 3;11:96. doi: 10.1038/s41467-019-13661-w (PMC6941990; doi:10.1038/s41467-019-13661-w)
Supplement: Supplementary file 1 — Supplementary Information [file 41467_2019_13661_MOESM1_ESM.pdf]

Supplementary Information for:

A wind-albedo-wind feedback driven by landscape evolution

Abell et al., 2019

Supplementary Note 1

Discussion of Model Validation

In order to provide validation for the use of the WRF model over the western Gobi region to interrogate our hypotheses, we compare results from WRF_Control to both global reanalysis (using Domain 1) and ground-based single-station observational datasets (using Domain 2) (Supplementary Figure 2). Here, we expand upon the results presented within the main text, and discuss some possible explanations for discrepancies found between the WRF_Control results and the observations.

As previously mentioned, WRF_Control does well in reproducing T2, SP, and 500-Winds from the data used to force the model (GFS-ANL) and an independent reanalysis product (ERA5)[1]. Generally, the WRF model does tend to overestimate T2 and 500-Winds, while also underestimating SP. In addition, the control simulation shows a positive bias in 10-Winds for all months. The larger disagreement for this variable (when compared to T2, SP, and 500-Winds) could arise from the effects of topography in the higher resolution WRF model compared to the reanalysis datasets. To go beyond a visual, qualitative comparison of the model output to the reanalysis datasets and provide statistical estimates of agreement/disagreement, the higher-resolution WRF_Control domain, which had a resolution of approximately ~0.3° x 0.3°, was coarsened to the lower-resolution 0.5° x 0.5° grids of the GFS and ERA5 datasets (see Methods of main text for further details). This process of re-gridding likely averaged higher surface winds near steep topography, creating higher near-surface wind speeds for the lower-resolution WRF_Control simulation than those found in ERA5 or GFS-ANL. This does not mean that the control run is incorrect, as it may be representing places of varying topography better than reanalysis data. One region where this becomes apparent is near Urumqi, Xinjiang, where areas

at the base of mountain ranges such as Dabancheng have yearly average wind speeds of ~6 m s^{-1} [3]. The control simulation correctly shows these higher wind speeds, but ERA5 and GFS do not (Supplementary Figure 4). Another possible cause for the higher surface wind speeds is the feedback between the higher resolution inner domains (Domains 2 and 3) with the outer domain (Domain 1) being used for this comparison. This is an effect of the nested domain configuration within the WRF model, and must be considered.

In addition to reproducing large-scale meteorology, it is critical to evaluate the ability of the model to reproduce small-scale, local conditions. To do this, daily-averaged observations from 14 sites that fall within Domain 2 of our model were compiled for the spring of 2011 (Supplementary Table 3; Supplementary Figure 5). Observational data are from the Integrated Surface Hourly dataset collected by USAF Climatology Center[2]. The output from WRF_Control was then averaged over a ~0.1° x 0.1° box surrounding the individual station's coordinates, and 24-hour individual time steps were averaged for each day to produce a final value. Again, while the WRF_Control simulation did well at reproducing T2, SP, and 10-Winds, it is important to mention the role of topography in trying to understand 1) the higher 10-Winds derived from the model and 2) the poor reproducibility for 6 of the stations. A majority of the days for 9 of the 14 stations show higher wind speeds derived from the model than the observations. Additionally, 6 of the 14 stations have less than 30% of the variance in their spring daily 10-Winds observations captured by the model. Due to the averaging over a specific 0.1° x 0.1° box, the inclusion of high wind speeds from mountain ranges would potentially explain the faster surface winds if the site was near steep topography (Supplementary Figure 6). It has been previously recognized that WRF can experience issues when simulating atmospheric conditions over small-scale topographic features[4,5]. The discrepancy between the model results and observations could also

be due to topography, or could reflect the effects of the nesting configuration mentioned above. We consider the model's ability to capture the trend, mean, and variability in spring near-surface wind speeds to be more important than the absolute values at any given day. WRF_Control is able to do this for over half of the stations examined.

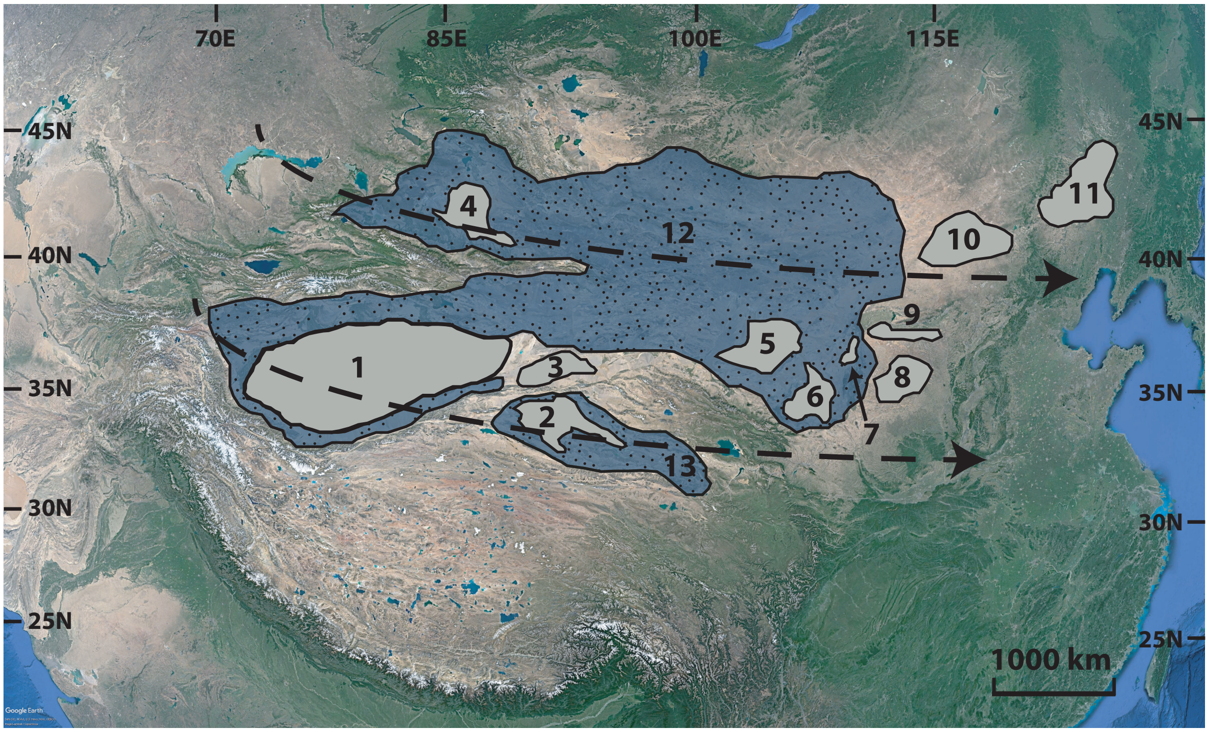

Supplementary Figure 1: Map of sandy and stony desert regions in East Asia. The light gray areas signify predominantly sandy deserts while the dark blue dotted regions are representative of stony deserts. 1: Taklimakan desert; 2, 13: Qaidam desert; 3: Kumtaq desert; 4: Gurban Tonggut desert; 5: Badain Jaran desert; 6: Tengger desert; 7: Ulan Buh desert; 8: Mu Us sandy land; 9: Qubqi desert; 10: Otindaq sandy land; 11: Horqin sandy land; 12: Gobi Desert. Arrows show possible westerly track during both glacial (lower) and interglacial (upper) periods[6]. Desert distributions and names are adapted from Laurent et al., 2005[7]; Sun et al., 2001[8]; Fullen and Mitchell, 1994 (copyright holder: The Royal Swedish Academy of Sciences)[9]. Created using Google Earth – US Department of State Geographer, ©2018 Google, ©2018 ZENRIN, Image Landsat/Copernicus.

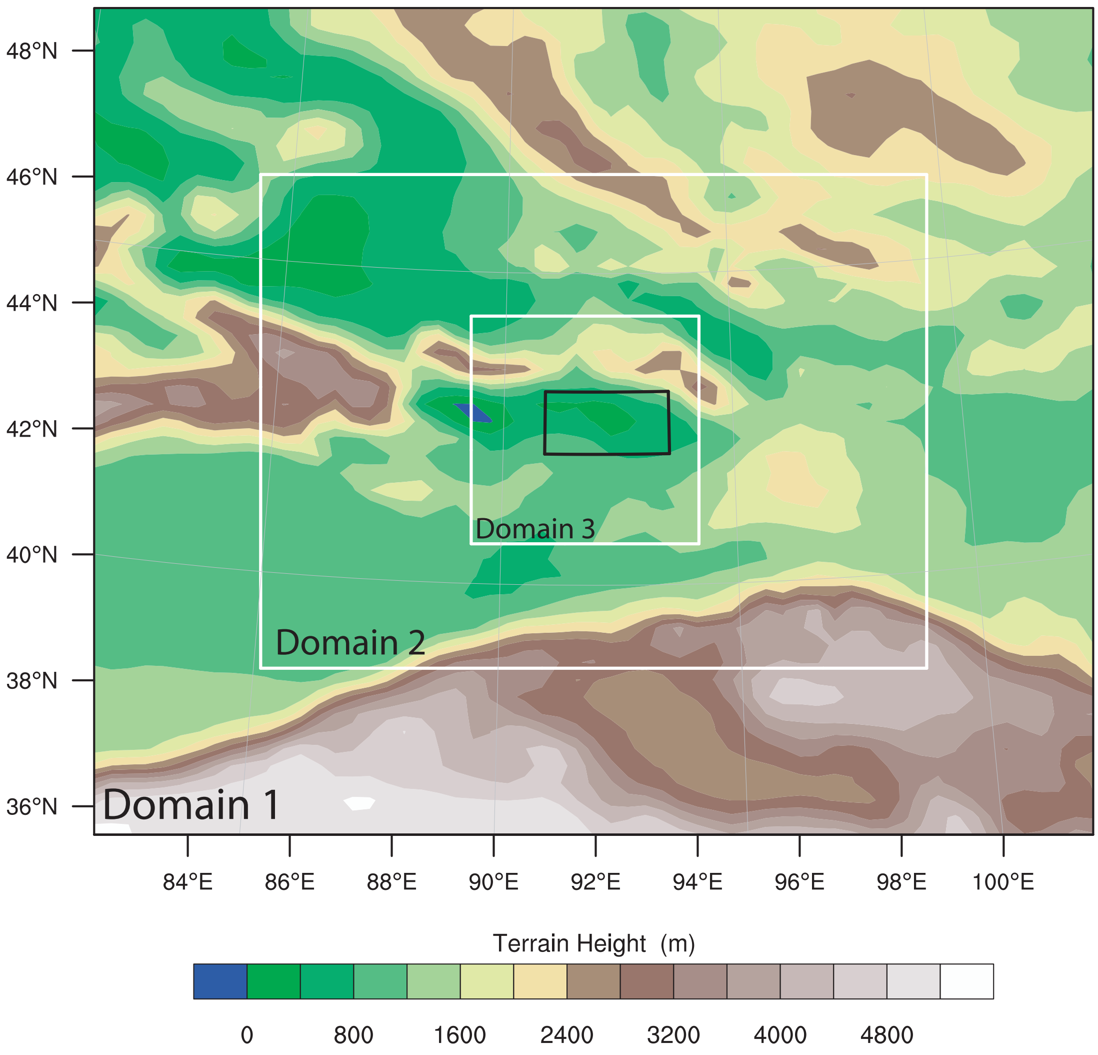

Supplementary Figure 2: Map of the three domains used for the WRF model simulations. Increasing domain number signifies increasing resolution for the model simulation. The outer (Domain 1 – D01), middle (Domain 2 – D02), and inner (Domain 3 – D03) had horizontal grid spacings of 30 km, 10 km, and 3.33 km, respectively. The black box indicates the approximate region where the albedo was altered in each run.

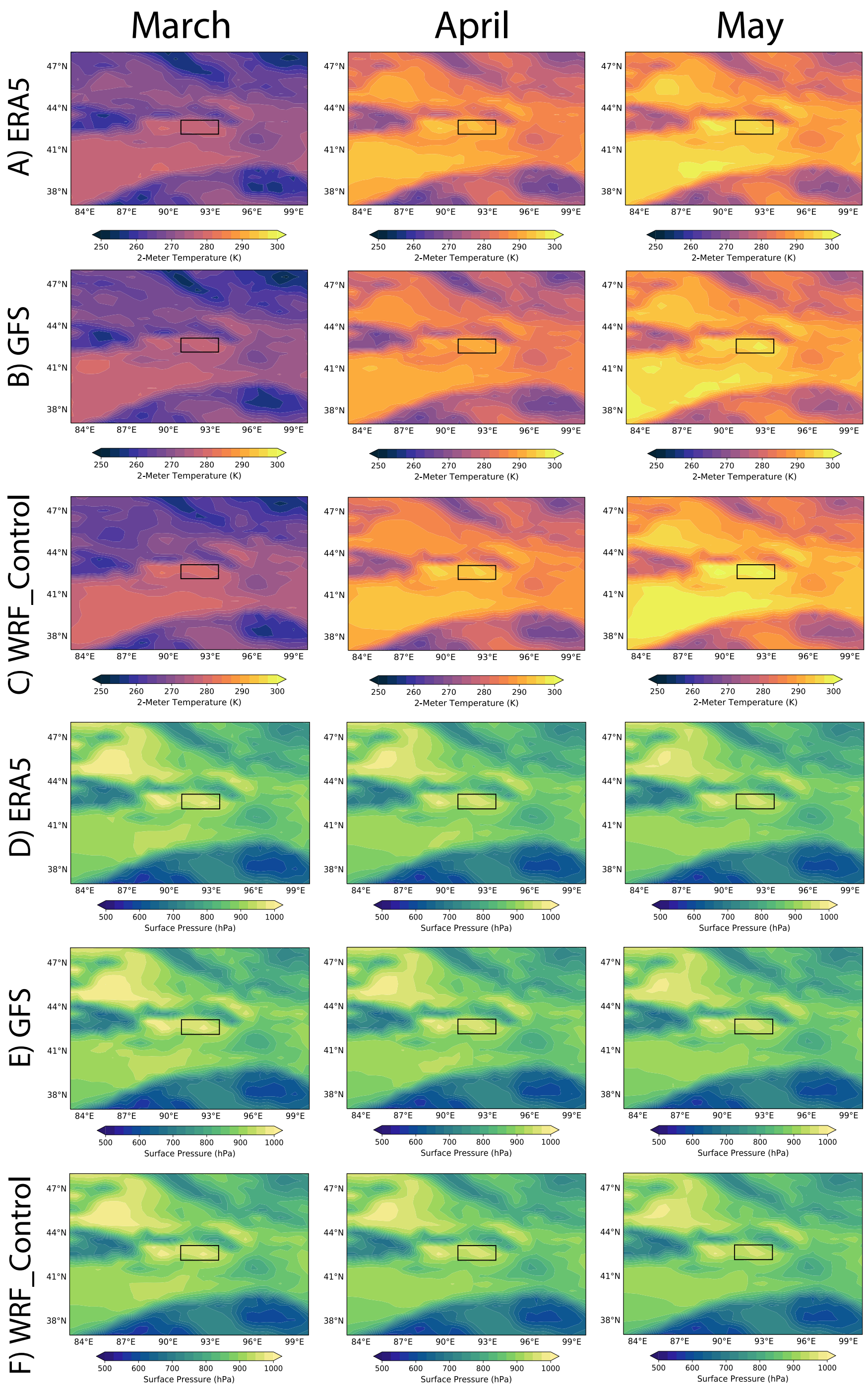

Supplementary Figure 3: Monthly 2-m temperature and surface pressure for model and reanalysis output for the spring of 2011. (A) Monthly 2-m temperature (K) for ERA5. **(B)** Same as A, but for GFS-ANL. **(C)** Same as A, but for the control simulation (WRF_Control). **(D)** Monthly surface pressure (hPa) for ERA5. **(E)** Same as D, but for GFS-ANL. **(F)** Same as D, but for WRF_Control.

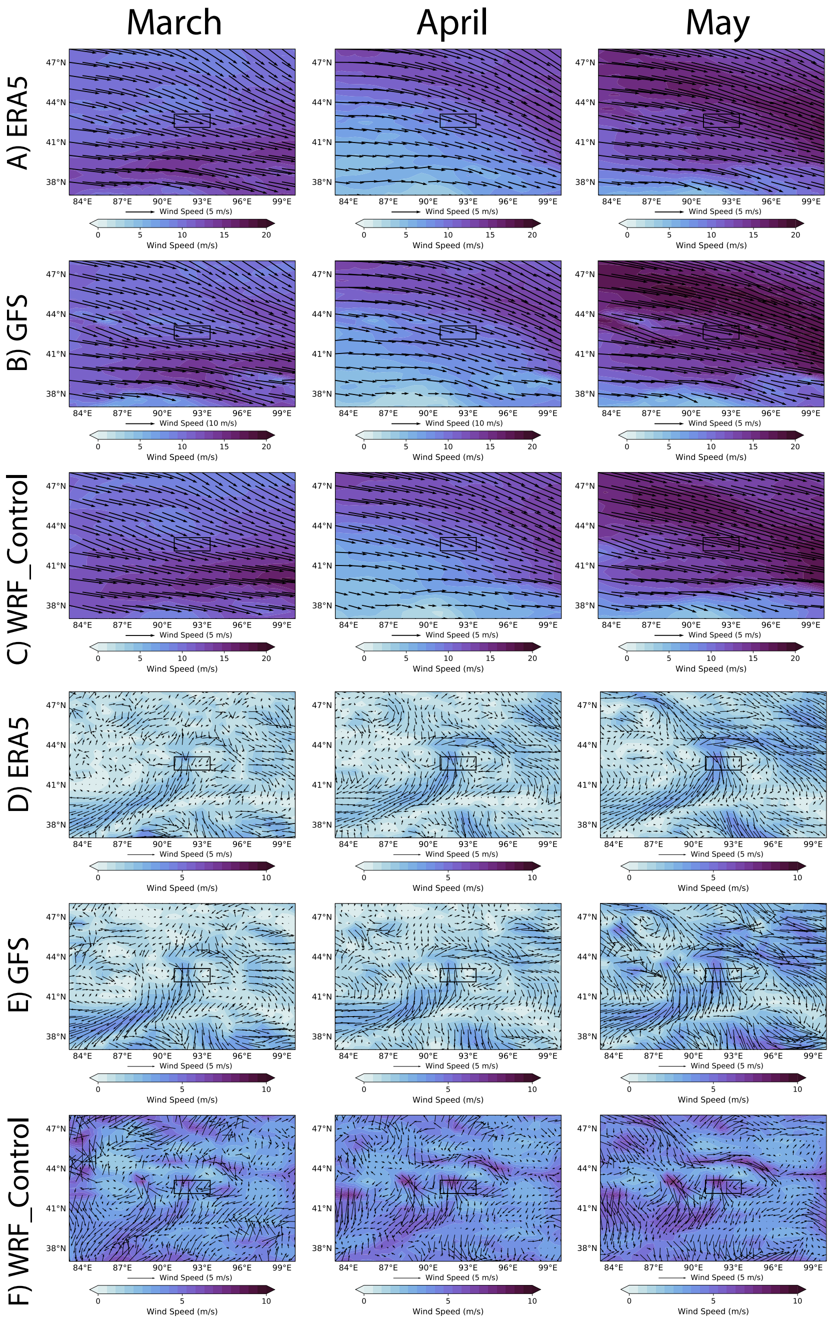

Supplementary Figure 4: Monthly 500-mb and 10-m horizontal wind speeds and direction for model and reanalysis output for the spring of 2011. (A) Monthly 500-mb horizontal wind speeds (m s^{-1}) (shading) and vectors for ERA5. **(B)** Same as A, but for GFS-ANL. **(C)** Same as A, but for the control simulation (WRF_Control.) **(D)** Monthly 10-m horizontal wind speeds (m s^{-1}) (shading) and vectors for ERA5. **(E)** Same as D, but for GFS-ANL. **(F)** Same as D, but for WRF_Control.

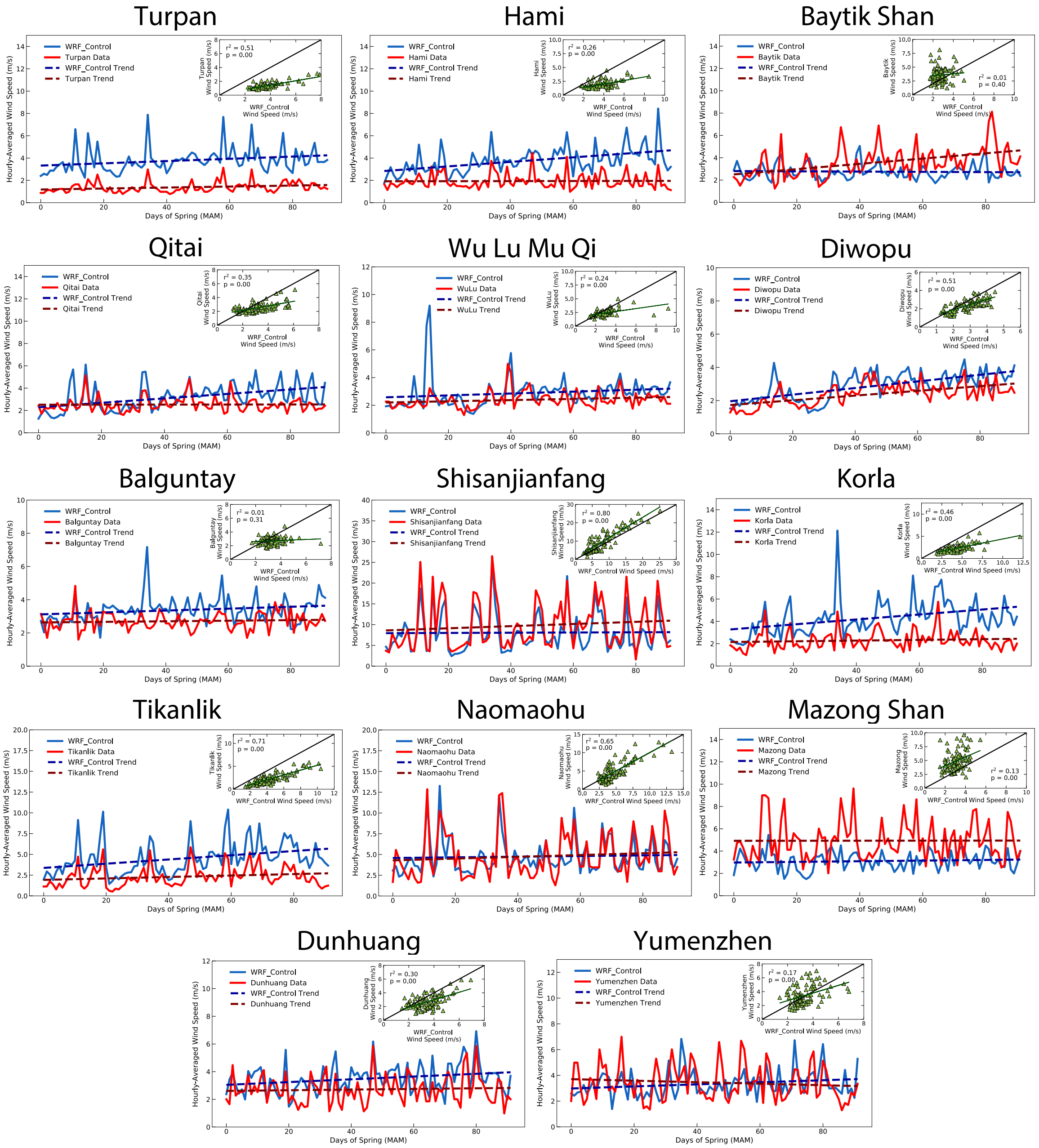

Supplementary Figure 5: Control simulation (WRF_Control) and station observations of 10-m horizontal wind speeds at 14 locations within Domain 2 for the spring of 2011. Lines represent daily averaged 10-m wind speeds for the control simulation (WRF_Control) (averaged over a 0.1° x 0.1° box surrounding the station) and ground-based observations over the spring (March, April, and May) of 2011. Dotted lines indicated trends through time. Insets are scatter plots of the points within the larger plots. r^2 and p-values are presented in the smaller plots.

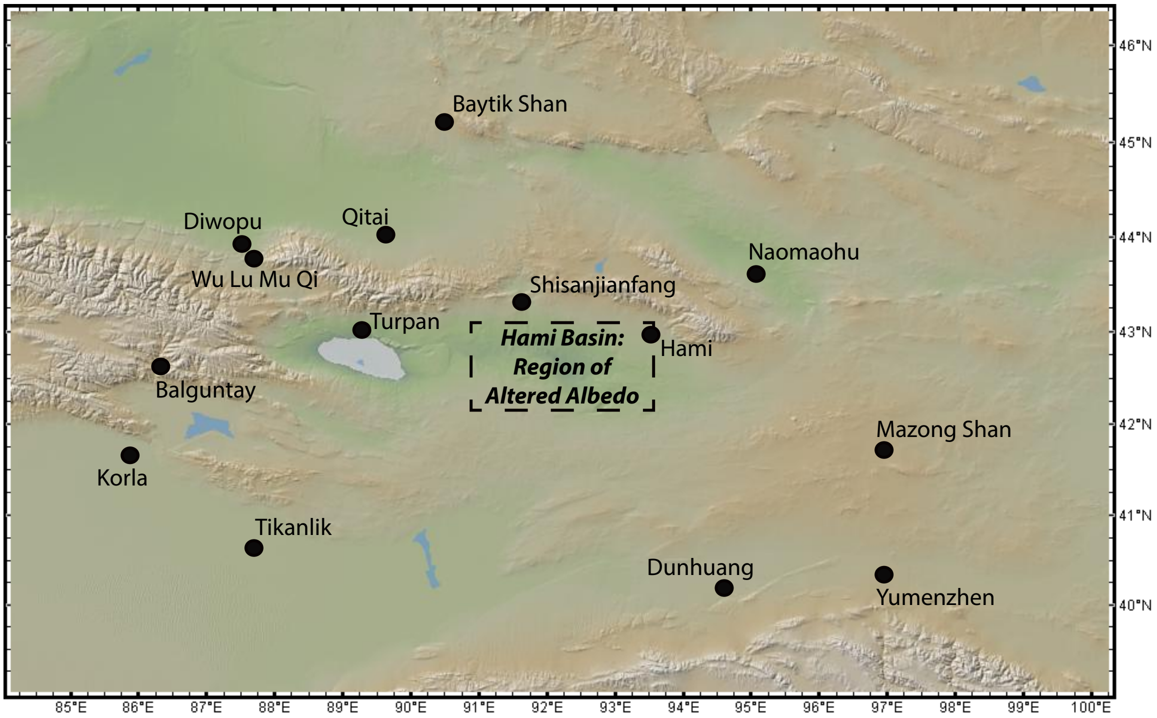

Supplementary Figure 6: Locations of ground-based observation stations used in model validation. Dotted box indicates the approximate regions where the albedo was altered for the simulations. Figure made with GeoMapApp (www.geomapapp.org) / CC BY / CC BY[10].

Supplementary Table 1: Descriptions of model simulations.

Simulation	Albedo (%)	All other variables
WRF_Control	Control (~12-15% throughout Hami)	Control
Albedo_50	50%	Control
Albedo_40	40%	Control
Albedo_30	30%	Control
Albedo_20	20%	Control
Albedo_10	10%	Control

Supplementary Table 2: Monthly statistical metrics for the control simulation (WRF_Control) output for Domain 01 against reanalysis data.

	GFS-ANL (0.5x0.5º)				ECMFW-ERA5 (0.5x0.5º)		
	**March**	**April**	**May**		**March**	**April**	**May**
				**2-Meter Temperature (K)**			
**MBE**	1.09	2.11	1.21		0.50	0.71	1.50
**MAE**	1.76	2.19	1.44		1.77	1.21	1.61
**RMSE**	2.12	2.51	1.71		2.25	1.51	1.86
				**Surface Pressure (hPa)**			
**MBE**	-0.32	-0.78	-0.81		0.35	-0.22	-0.43
**MAE**	5.41	5.35	5.29		4.65	4.40	4.34
**RMSE**	7.82	7.68	7.58		6.77	6.49	6.44
				**500mbar Wind Speed (m/s)**			
**MBE**	0.34	0.09	-0.85		0.26	0.05	0.11
**MAE**	0.83	0.64	1.08		0.70	0.50	0.66
**RMSE**	1.16	0.86	1.34		0.94	0.66	0.83
				**10-Meter Wind Speed (m/s)**			
**MBE**	2.32	2.49	1.76		2.47	2.60	2.44
**MAE**	2.32	2.50	1.90		2.47	2.60	2.47
**RMSE**	2.60	2.71	2.22		2.68	2.79	2.73

Supplementary Table 3: Spring (March, April, May) 2011 average statistical metric for the control simulation (WRF_Control) output for Domain 02 against observational station data.

	Turpan	Hami	Baytik	Qitai	WuLu	Diwopu	Balguntay	Shisanjianfang	Korla	Tikanlik	Naomaohu	Mazong	Dunhuang	Yumenzhen
							**2-Meter Temperature (K)**							
**MBE**	2.25	2.54	2.25	4.23	1.56	1.05	-2.67	0.68	2.55	3.53	1.43	2.94	3.56	3.67
**MAE**	2.37	2.61	2.64	4.23	1.99	2.05	2.74	1.01	2.59	3.54	1.61	2.98	3.56	3.67
**RMSE**	2.77	3.07	3.03	4.89	2.43	2.42	2.98	1.28	2.97	3.99	1.90	3.28	3.76	3.88
**r^2**	0.97	0.97	0.96	0.97	0.98	0.97	0.98	0.99	0.97	0.96	0.98	0.97	0.98	0.98
**p-value**	0.00	0.00	0.00	0.00	0.00	0.00	0.00	0.00	0.00	0.00	0.00	0.00	0.00	0.00
							**Surface Pressure (hPa)**							
**MBE**	-10.49	-5.04	8.40	N/A	2.37	N/A	-47.34	N/A	-0.37	-2.01	N/A	-1.76	-2.77	0.92
**MAE**	10.49	5.17	8.40	N/A	2.37	N/A	47.34	N/A	1.16	2.08	N/A	1.93	2.99	1.29
**RMSE**	10.63	5.30	8.48	N/A	2.59	N/A	47.37	N/A	1.79	2.36	N/A	2.15	3.20	1.71
**r^2**	0.97	0.90	0.94	N/A	0.98	N/A	0.88	N/A	0.94	0.97	N/A	0.93	0.94	0.93
**p-value**	0.00	0.00	0.00	N/A	0.00	N/A	0.00	N/A	0.00	0.00	N/A	0.00	0.00	0.00
							**10-Meter Wind Speed (m/s)**							
**MBE**	2.40	1.83	-0.83	0.67	0.49	0.49	0.67	3.14	2.00	2.20	-0.02	-1.83	0.78	-0.09
**MAE**	2.40	1.85	1.26	0.90	0.63	0.63	0.86	-1.71	2.00	2.20	1.22	1.94	1.03	1.08
**RMSE**	2.53	2.08	1.66	1.15	1.08	0.75	1.10	2.41	2.31	2.47	1.56	2.45	1.25	1.33
**r^2**	0.51	0.26	0.01	0.35	0.24	0.51	0.01	0.80	0.46	0.71	0.65	0.13	0.30	0.17
**p-value**	0.00	0.00	0.40	0.00	0.00	0.00	0.31	0.00	0.00	0.00	0.00	0.00	0.00	0.00

Supplementary Table 4: Schemes applied in WRF simulations.

Model Parameter	Scheme	Citations
Microphysics	Morrison 2-Moment Scheme	Morrison et al., 2009
Radiation	RRTMG LW and SW radiation schemes	Iacono et al., 2008
Planetary Boundary Layer	MYJ planetary boundary layer scheme	Janjić, 1994
Convection	Kain-Fritsch convection scheme	Kain, 2004
Surface Layer	Monin-Obukhov similarity theory	
Land Surface	Unified Noah Land Surface scheme	Tewari et al., 2004
Land-based variables	24-category U.S. Geological Survey (USGS) dataset	Loveland et al., 2000; Sertel et al., 2010
Climate Boundary Conditions	NCEP GFS-ANL 0.5° x 0.5°	NCEP

Supplementary References

1. Copernicus Climate Change Service (C3S): ERA5: Fifth generation of ECMWF atmospheric reanalyses of the global climate. Copernicus Climate Change Service Climate Data Store (CDS). Accessed August, 2019. https://cds.climate.copernicus.eu/cdsapp#!/home. (2017).

2. NOAA National Centers for Environmental Information: Global Surface Hourly Global Summary of the Day (GSOD). NOAA National Centers for Environmental Information. Accessed August, 2019. (2001).

3. Yao, Z., Xiao, J., and Jiang, F. Characteristics of daily extreme-wind gusts along the Lanxin Railway in Xinjiang, China. *Aeolian Research*. **6**, 31–40, (2012).

4. Jiménez, P. A., and Dudhia, J. On the ability of the WRF model to reproduce the surface wind direction over complex terrain. *Journal of Applied Meteorology and Climatology*, **52**, 1610-1617, (2013a).

5. Jiménez, P. A. et al., Analysis of the long-term surface wind variability over complex terrain using a high spatial resolution WRF simulation. *Climate dynamics*, **40**, 1643-1656, (2013b).

6. Kapp, P. et al. Wind erosion in the Qaidam basin, central Asia: implications for tectonics, paleoclimate, and the source of the Loess Plateau. *GSA Today*, **21**, 4-10, (2011).

7. Laurent, B. et al. Simulation of the mineral dust emission frequencies from desert areas of China and Mongolia using an aerodynamic roughness length map derived from the POLDER/ADEOS 1 surface products. *Journal of Geophysical Research: Atmospheres*, **110**, (2005).

8. Sun, J., Zhang, M., and Liu, T. Spatial and temporal characteristics of dust storms in China and its surrounding regions, 1960–1999: Relations to source area and climate. *Journal of Geophysical Research: Atmospheres*, **106**, 10325-10333, (2001).

9. Fullen, M. A., and Mitchell, D. J. Desertification and reclamation in north-central China. *Ambio*, 131-135, (1994). (copyright holder: The Royal Swedish Academy of Sciences).

10. Ryan, W. B. F., et al. Global Multi-Resolution Topography (GMRT) synthesis data set, *Geochem. Geophys. Geosyst.*, **10**, Q03014, (2009).
