# Supplementary material for: A wind-albedo-wind feedback driven by landscape evolution
Source: Nat Commun. 2020 Jan 3;11:96. doi: 10.1038/s41467-019-13661-w (PMC6941990; doi:10.1038/s41467-019-13661-w)
Supplement: Supplementary file 3 — Description of Additional Supplementary Files [file 41467_2019_13661_MOESM3_ESM.pdf]

Description of Additional Supplementary Files

File name: Supplementary Data 1

Description: Spring Model Variable Output for Hami Basin (Domain 3)
